# Molecular allergology and its application in prevention, diagnosis and therapy

**DOI:** 10.3389/falgy.2023.1260902

**Published:** 2023-08-07

**Authors:** Aleksandra Podzhilkova, Christoph Nagl, Karin Hoffmann-Sommergruber

**Affiliations:** Institute of Pathophysiology and Allergy Research, Center of Pathophysiology, Infectiology and Immunology, Medical University of Vienna, Vienna, Austria

**Keywords:** allergy, allergen diagnosis, allergens, IgE-mediated allergy, molecular allergology

## Abstract

Allergic diseases represent a relevant global health problem, affecting adults and children and posing a significant burden for health care systems. In addition, the disease is still under-recognized and harmonized diagnostic tools and management plans for patients are still lacking. In this review the most important aspects of the diagnosis of allergic diseases are summarized and the contribution of Molecular allergology to this area is highlighted.

## 1. Introduction

Allergic diseases affect almost 30% of the population worldwide and induce symptoms of the respiratory system, gastrointestinal tract, skin, and the cardiovascular system (1). Allergy is a result of IgE-mediated immune responses to a foreign, usually harmless, protein (allergen). Allergens, recognized by the immune system, can induce mild or severe symptoms up to even life-threatening reactions in both, atopic children and adults (2, 3).

The immune system plays a critical role to protect the human body from viral or microbial infections. However, sometimes overstimulation of the immune system can lead to the opposite effect and induce inflammatory responses, also known as hypersensitivity reactions, Type I-IV (4, 5). Allergic reactions of Type I hypersensitivity, are defined as an immediate reaction against harmless antigens, mostly proteins, resulting in the production of specific immunoglobulin E (IgE) antibodies. This antibody-antigen interaction leads to mast cells degranulation and release of histamine and other inflammatory mediators (4) and causes vasodilation, decrease of blood pressure, bronchoconstriction and in some cases—anaphylactic shock (6).

The allergic sensitisation can develop via different ways of exposure—by inhalation, ingestion, injection, and skin contact. Aeroallergens are associated with increasing cases of respiratory disorders such as allergic rhinitis and asthma (7, 8). House dust mites (HDM), moulds, and animal fur and dander particles are frequent indoor allergen sources, while tree, weed, and grass pollens are the leading outdoor allergen sources (7, 9, 10).

Birch and grass pollens are the most common causes of allergic rhinitis and asthma in Europe (11). However, during the flowering season, pollen exposure levels can vary due to atmospheric factors such as wind, humidity, and rainfall. In addition, anthropogenic climate changes may have an impact on flowering seasons and thus pollen loads (12).

Food allergies represent another relevant health problem affecting patients of all age groups. In Westernized countries, the percentage of estimated overall food allergies are about 5% in adults and 8% in children (13). For example, allergy to cow's milk affects approximately 2.5% of the infants during the first two years of life. However, the majority of those pediatric patients resolves their milk allergy at school age (14, 15).

Allergy to tree nuts and peanuts can develop at any age. In Europe hazelnut sensitisation rates range from 1.8% (Reykjavik) to 14.35% in Zurich. However, the probable food allergy prevalence is much lower but differing across Europe, ranging from 0.06% for Athens vs. 2.57% in Zurich (16). Similar rates were observed for walnut allergy. An Australian study reported a prevalence of 2.3% of clinically relevant tree nut allergy with cashew and pistachio nuts being the most frequent allergenic tree nuts. Peanut allergy affects 1%–3% of children and persists throughout lifetime (17). The symptoms range from eczema, urticaria up to asthma and anaphylaxis. Sensitization to peanut allergens Ara h 5 and Ara h 8, belonging to the PR10 protein family and profilin, respectively usually induces symptoms belonging to the oral allergy syndrome. In contrast, Ara h 1, Ara h 2, and Ara h 3, belonging to the seed storage proteins, tend to induce more severe and systemic reactions (16, 18, 19).

Despite the great amount of allergens that are already described, the characterization of allergens, particularly in regions like Asia and Africa, are still missing due to lack of comprehensive data on allergens prevalent in local environments and food sources. Future campaigns including increased funding for joint projects, awareness campaigns and knowledge sharing initiatives can help to improve allergen characterization in Asian and African countries, leading to better allergy management and public health outcomes in these regions.

### 1.1. What is an allergen?

Allergens are typically proteins recognized by the immune system from a predisposed individual as foreign and potentially harmful. Upon contact with an allergen, the immune system produces specific antibodies called immunoglobulin E (IgE) antibodies (2).

Proteins, sharing similar sequences and structural elements, are assigned to protein families. Currently around 19,000 protein families are described. Out of those approximately 40 protein families contain allergenic proteins. Sequence similarity and shared structural elements of allergens are responsible for IgE cross reactivities (16).

In the recent past, bioscience has tremendously improved due to up to date technology which enables precise characterization of proteins including allergens. This approach allows to identify and produce well defined highly pure molecules for component resolved diagnosis which in turn facilitates a patient tailored diagnosis and management. Some examples of the application of “Molecular Allergology” are described below.

Depending on the allergen source, there are different types of allergens present which can cause symptoms ranging from mild to severe reactions. For example, in birch pollen several allergens have been identified. Among those, Bet v 1 is the major allergen belonging to the pathogenesis-related protein family PR-10 (20). In an Austrian study of 501 adolescents, 16.3% showed IgE reactivity to Bet v 1, and in Switzerland and Denmark, the prevalence of birch pollen sensitization was 7.9% and 13.7%, respectively (21–23). Bet v 1 can exhibit cross-reactivity with highly homologous proteins present in a range of other allergen sources including pollens and plant foods. For example, individuals allergic to Bet v 1 may experience oral allergy syndrome when consuming certain fruits and vegetables, such as apple (Mal d 1), hazelnut (Cor a 1), peach (Pru p 1), and carrot (Dau c 1) due to cross-reactivity between Bet v 1 and similar proteins in these foods (24–26).

Another important type of allergen that causes allergic reactions is the cupin superfamily, usually present in allergenic food sources, such as tree nuts, peanuts and soybeans. Cupins (11S and 7S globulins) are characterized by their barrel-shaped structure and play various biological roles in plants, such as storage of nutrients, and defence mechanisms against pathogens (27). The most abundant nut allergens are seed storage proteins: vicilins (7S trimeric globulins), and legumins (11S hexameric globulins), followed by 2S albumins, nsLTPs, profilins and hevein-related proteins (28). Cross-reactivity among cupins from different plant sources can occur (29). For example, if someone is allergic to peanut cupin, they may also experience allergic reactions when consuming other legumes or seeds that contain similar cupin proteins, such as soybeans or sesame seeds. This cross-reactivity is due to the shared structural characteristics and sequence similarities among these proteins which are recognized by specific IgE molecules.

Non-specific lipid transfer proteins (nsLTPs) have been identified from various plant sources, including fruits, vegetables, nuts, and seeds (30). These proteins form a cavity which binds and transfers lipids (fatty acids and phospholipids) across cell membranes (31). While nsLTPs serve important functions in plants, they are also recognized as allergens in certain individuals. Due to the similarities in protein structures and sequences among nsLTPs from different plant sources, individuals sensitized to a given nsLTP from one food, may also experience allergic reactions when consuming other foods that contain similar nsLTPs. For example, sensitization to peach nsLTP (Pru p 3) may result in cross-reactivity with nsLTPs from other fruits like apple (Mal d 3), cherry (Pru p 3), or hazelnut (Cor a 8) (30, 32).

Recently, novel allergens present in fruits and pollens were identified as Gibberellin-regulated proteins (GRPs). The physicochemical properties of these proteins overlap to some extent with nsLTPs. Gibberellin-regulated proteins are small cationic proteins, contain 6 conserved disulfide bridges and are expressed in pollen, peel and pulp of fruits. They are upregulated upon pathogen attack. Allergens have been reported from peach, apricot, cherry and citrus fruits and from Cupressaceae pollen (16). Based on their structure these proteins also contain a cavity. However, no specific ligands have been identified so far.

Despite the increasing knowledge of different allergenic protein families including their sequences and structural determinants, it still remains unclear why certain proteins are inducing an allergic reaction. Recent studies provided evidence that additional compounds such as lipids, carbohydrates, and fibres may contribute to the allergic sensitization and effector phase (33).

For example, Bet v 1, nsLTP, 2S albumins, secretoglobins, and lipocalins contain hydrophobic binding sites for lipid ligands which can enhance a Th2-response (33–35). In a recent study by Janssen-Weets et al. the ligand binding activity of mammalian lipocalins and secretoglobins were investigated and fatty acids, fatty alcohols, and the terpene alcohol farnesol were the most relevant ligands with strong binding affinities to the proteins. These ligands may contribute to the immunomodulating activity of allergens and thus to allergic sensitization, although further studies are needed to prove this hypothesis (7).

Tree nuts have a high lipid content, ranging from 46%–83% of fatty acids, depending on the nut source (35, 36). Recently it was shown (8, 35, 36) that lipids bound to proteins can decrease the epithelial barrier integrity thereby contributing to the allergic sensitization process. This in turn facilitates the tight interaction of lipids with several components of the innate immune system (34, 37, 38).

Also, glycan moieties can act as immunomodulating compounds. Glycosylated allergenic proteins and peptides are interacting with specific receptors on dendritic cells, such as the macrophage mannose receptor, thus inducing an (innate) immune response (21).

Furthermore, allergens usually come together with additional substances such as pollen compounds, or food matrix components. Simultaneous exposure to both allergens and potent immunogenic substances may thus facilitate the onset of an allergic sensitization process.

### 1.2. Diagnosis of IgE mediated allergy

**Figure 1:**
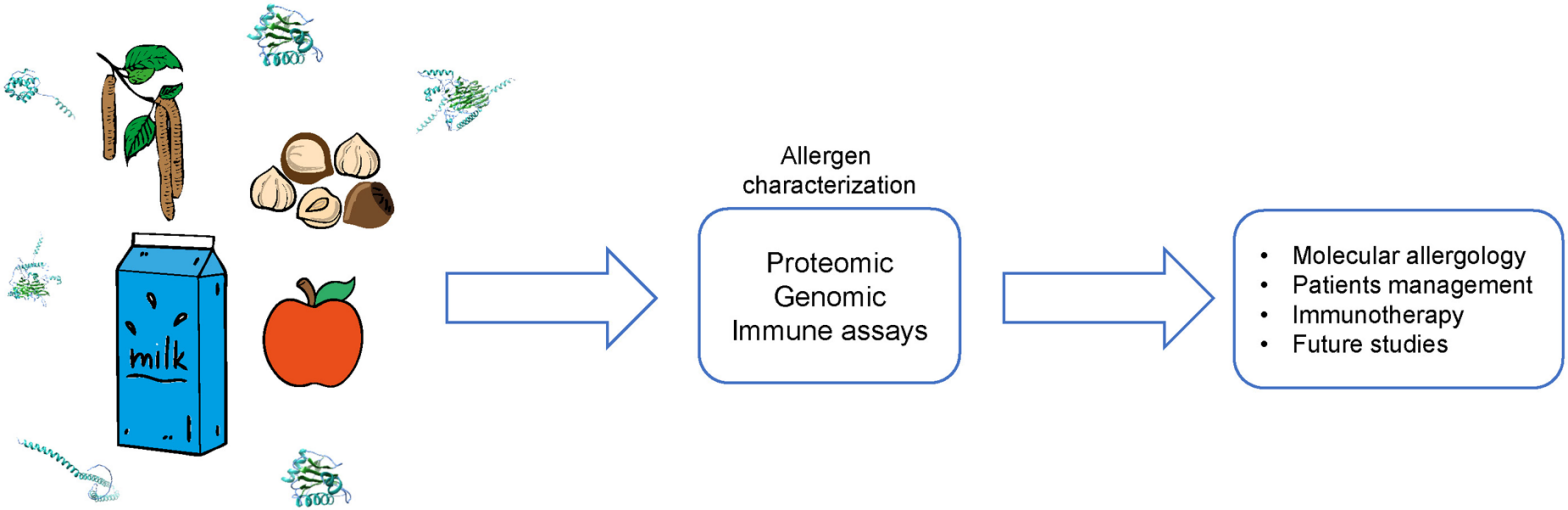
Overview on the critical steps of molecular allergy diagnosis.

The diagnostic procedure for IgE-mediated allergy usually starts with a detailed assessment of the patient's history, followed by different tests: skin prick test, basophil activation test, serum-specific IgE tests, and food challenge tests to identify the range of IgE sensitisation (Figure 1). For a restricted number of patients challenge tests are performed such as pollen exposure or food challenge tests.

Allergy diagnostics aim to identify the culprit allergen source and to measure the specific IgE values. In the following some of the commonly used tests are summarised:

1. Skin Prick Test (SPT): In this method, a small amount of allergen extract is applied to the skin, usually on the forearm or back. The skin is then pricked with a sterile lancet, allowing the allergen to enter the skin. If the person is allergic to a particular allergen present in the extract, they will develop a local reaction (“wheal and flare”), within 15–20 min. Positive control (histamine) and negative control (NaCl) are run in parallel. Skin prick tests identify reactions to inhalants, foods, some drugs, occupational allergens, hymenoptera venom and latex (39). Sometimes prick to prick tests are used to test a potential allergen source directly on the skin. The limitations of SPTs include:
  • False positive or false negative results;
  • Potential for allergic reactions: while the test is generally safe, there is a small risk of severe allergic reactions (anaphylaxis) in highly sensitive individuals. Skin prick tests should always be performed under the supervision of a qualified healthcare professional who can promptly manage any adverse reactions (40);
  • In some cases, skin prick tests may produce inconclusive results. This can occur when the skin reacts to multiple allergens, making it challenging to determine the primary trigger. False negative results may be due to the fact the allergen extracts miss the culprit allergen: Additionally, individuals with certain skin conditions, such as eczema or other skin diseases, may have difficulty interpreting the test results accurately.
2. Several serum-specific IgE tests are currently used in clinical routine. Allergen extracts or single allergen batches are coupled to a solid phase and after serum incubation bound IgE antibodies are detected by labeled anti-IgE antibodies allowing a quantitative and qualitative analysis. While allergen extract based tests are regarded as a primary approach, chip based test formats containing more than hundred individual allergens are also available. The first approach provides information on the culprit allergen source and has a generally higher test sensitivity. Limitations of this approach are the batch to batch variation and the potential lack of individual allergens in some sources and the required higher volume of serum samples. The second approach also called multiplex testing provides information on IgE sensitization to individual allergens. This test has a higher specificity and requires only minute amounts of serum samples. Limitations of this test format are higher costs and less availability on a global scale. While both tests provide information on IgE sensitization to allergen sources, the multiplex assay allows to assess the allergen specific sensitization pattern, differentiating between cross-reactive structures and species specific allergens. Furthermore, information on sensitization to marker allergens allows a detailed risk assessment regarding disease progression and personalized treatment options (16).
3. The Basophil Activation Test (BAT) provides information on the presence of allergen specific IgE and cellular activation and degranulation (16). Due to the activation of basophils by allergen extracts or specific allergens, upregulation of surface marker expression can be measured and allows to assess the onset of an allergic reaction (41, 42). BAT assays can be performed using either patient-derived cells or by applying a standardized cell population from a given donor. In this case, basophils are then incubated with serum samples from patients (also called passive sensitization). Recently, Stoffersen et al. reported that the required spIgE levels for optimal cellular activation are ranging from 3,51 kUA/L (CAP class 3) up to >17,5 kUA/L (CAP class 4) using Bet v 1 as a model (43). BAT represents a promising approach for diagnosing allergic reactions (43). This functional test provides a quick and reliable way to identify specific allergens using patients' samples, paving the way for more effective treatments and personalized care. In specialized centers BAT is already included in daily diagnosis of allergies. Especially for peanut and sesame allergy a BAT assay is recommended if other test results remain inconclusive (9, 41, 42). Wheat dependent exercise-induced anaphylaxis (WDEIA) is a co-factor induced wheat allergy and considered to be a rare disease. Gabler and co-authors tested a range of gluten and non-gluten proteins from wheat in the BAT assays using samples from WDEIA patients. Using this approach they could identify gluten and additional non-gluten proteins from wheat as allergens relevant for WDEIA. This approach may improve the diagnosis of this disease and help to rule out idiopathic anaphylaxis for these patients (41). Recently, BAT tests have been shown to be helpful in another IgE mediated disease, ABPA, allergic bronchopulmonary aspergillosis. This disease is caused by the ubiquitous mould *Aspergillus fumigatus* and may be underdiagnosed in certain patient groups suffering from a chronic pulmonary disease. Michel and coauthors showed that BAT assays performed with samples from a cohort of patients proved useful in the diagnosis for ABPA, when using *A. fumigatus* extract and Asp f 1, 2, 3, 4, 6, respectively (9).

### 1.3. Molecular allergology and its application in prevention and management of the patient

As mentioned above molecular allergology has improved diagnosis of allergies tremendously. However, for molecular testing the quality of the analytes, that is highly pure and well characterized batches of natural or recombinant allergens, is crucial. This approach requires powerful expression platforms for recombinant proteins and purification procedures for both natural and recombinant proteins, respectively. Critical steps of the final quality assessment of allergen batches include purity, intact tertiary protein structure, and biological activity. To assess these parameters a number of physicochemical methods can be applied including mass spectrometry, CD spectroscopy, and NMR analyses. In parallel the IgE binding activity needs to be confirmed.

While for some allergens only single isoforms have been identified, other proteins are present as a range of different isoforms. To identify a representative isoform of diagnostic relevance several analytical methods need to be applied. Recently Marsh and colleagues performed a study on the range of isoforms of peanut allergens Ara h 1, 2, 3, and 6 to assess the range of isoforms present in 20 different peanut genotypes. Applying LC-MS/MS and RP/HPLC they could quantify and compare the content of Ara h 1,2,3, and 6 present in various commercially used genotypes. This approach is important for food allergy risk assessment and can be applied to obtain well defined reference material to be used for allergen detection (27).

Various physicochemical parameters such as heat treatment or pH environment can affect the structure of an allergenic protein. This in turn may impact on its immunogenic potency (44). Using profilin as a model protein family, pollen allergens Amb a 8, Art v 4, and Bet v 2 were selected and a MD simulation strategy applied to assess their structural dynamics at varying pH levels. Although these proteins share high structural and sequence similarities they showed different fold stability under different pH conditions (44).

With the application of molecular allergology the immunological effects during specific immunotherapy can also be assessed. Thörnqvist and colleagues used a peptide based approach to assess the development of specific antibody responses during grass pollen immunotherapy (45). An allergome-wide peptide microarray was developed and the IgG, IgE and IgG4 levels of grass pollen specific peptides were investigated using this high-throughput approach. The evolution of linear epitope-specific antibody responses were analysed. These data contribute to our current understanding of the development of the immune response during immunotherapy and can help to further improve immunotherapeutics.

In summary the application of molecule based allergy diagnosis provides additional information regarding allergens, helps to assess the range of potential cross reactivities, and for some allergen families also the severity of symptoms caused. This information can further help to give personal advice to patients, and train them to avoid exposure to the allergen sources such as pollens, plant foods and their presence in processed foods.

## 2. Conclusions

Allergy diagnostics help in accurately identifying the specific allergens that trigger an individual's allergic reactions. This knowledge is crucial for understanding the underlying cause of allergies and providing targeted and personalized treatment plans. Once the allergens are identified through diagnostic testing, healthcare professionals can develop effective treatment strategies. This may include allergen avoidance measures, medication management, or allergen-specific immunotherapy (45–47). It can significantly improve the quality of life for individuals with allergies by reducing symptoms, fine-tuning medication plans, and potentially preventing the progression of allergic symptoms.
